# *Enterococcus faecalis* from Food, Clinical Specimens, and Oral Sites: Prevalence of Virulence Factors in Association with Biofilm Formation

**DOI:** 10.3389/fmicb.2015.01534

**Published:** 2016-01-11

**Authors:** Annette C. Anderson, Daniel Jonas, Ingrid Huber, Lamprini Karygianni, Johan Wölber, Elmar Hellwig, Nicole Arweiler, Kirstin Vach, Annette Wittmer, Ali Al-Ahmad

**Affiliations:** ^1^Department of Operative Dentistry and Periodontology, Center for Dental Medicine, Medical Center, University of FreiburgFreiburg, Germany; ^2^Department of Infection Control and Hospital Epidemiology, Institute for Environmental Health Sciences and Hospital Infection Control, Medical Center, University of FreiburgFreiburg, Germany; ^3^Bavarian Health and Food Safety AuthorityOberschleissheim, Germany; ^4^Department of Periodontology, Philipps-University of MarburgMarburg, Germany; ^5^Department of Medical Biometry and Statistics, Center for Medical Biometry and Medical Informatics, Medical Center, University of FreiburgFreiburg, Germany; ^6^Department of Medical Microbiology and Hygiene, Center for Microbiology and Hygiene, Medical Center, University of FreiburgFreiburg, Germany

**Keywords:** enterococci, virulence factors, biofilm formation, oral cavity, PFGE, antibiotic susceptibility

## Abstract

Enterococci have gained significance as the cause of nosocomial infections; they occur as food contaminants and have also been linked to dental diseases. *E. faecalis* has a great potential to spread virulence as well as antibiotic resistance genes via horizontal gene transfer. The integration of food-borne enterococci into the oral biofilm *in-vivo* has been observed. Therefore, we investigated the virulence determinants and antibiotic resistance of 97 *E. faecalis* isolates from the oral cavity, food, and clinical specimens. In addition, phenotypic expression of gelatinase and cytolysin were tested, *in-vitro* biofilm formation was quantified and isolates were compared for strain relatedness via pulsed field gel electrophoresis (PFGE). Each isolate was found to possess two or more virulence genes, most frequently *gelE, efaA*, and *asa1*. Notably, plaque/saliva isolates possessed the highest abundance of virulence genes, the highest levels of phenotypic gelatinase and hemolysin activity and concurrently a high ability to form biofilm. The presence of *asa1* was associated with biofilm formation. The biofilm formation capacity of clinical and plaque/saliva isolates was considerably higher than that of food isolates and they also showed similar antibiotic resistance patterns. These results indicate that the oral cavity can constitute a reservoir for virulent *E. faecalis* strains possessing antibiotic resistance traits and at the same time distinct biofilm formation capabilities facilitating exchange of genetic material.

## Introduction

Enterococci, Gram-positive, facultative anaerobic cocci, are resilient by nature and able to survive a wide array of hostile conditions and can persist in the environment for long periods of time (Van Tyne and Gilmore, 2014). They are known as commensals in the gastrointestinal tract and also play an important role in food ripening and the development of specific aromas of various cheeses and can cause spoilage of certain meats (Franz et al., 2011; Hammerum, 2012).

The two species *Enterococcus faecalis* and *Enterococcus faecium*, with the former being predominant, have gained significance in recent decades as leading opportunistic pathogens causing nosocomial infections (Murray, 1990; Hidron et al., 2008; Hammerum, 2012; Van Tyne and Gilmore, 2014). They are associated with various infections, including urinary tract infections, bacteraemia, meningitis, wound infections and neonatal infections. More recently, biofilm-associated infections of artificial medical devices have been attributed to enterococci (Sandoe et al., 2003; Arias-Moliz et al., 2012; Paganelli et al., 2012). These are difficult to treat due to increased antibiotic resistance in the biofilm. Furthermore, multi-drug resistant strains have complicated the treatment of these infections. Resistance to various antibiotics is common in enterococci (Arias and Murray, 2008) and vancomycin-resistant enterococci have been on the rise since the 1980s (Hammerum et al., 2004; Gilmore et al., 2013).

Apart from nosocomial infections, *E. faecalis*, although not normally considered to be part of the healthy oral flora (Aas et al., 2005), has been found in common dental diseases, i.e., periodontitis, periimplantitis and caries (Kouidhi et al., 2011; Dahlen et al., 2012; Rams et al., 2013). *E. faecalis* has been found primarily in secondary endodontic infections with a prevalence of 24–70%, i.e., in previously filled root canals, where it can also form biofilms (Siqueira and Rocas, 2009; Vidana et al., 2011; Anderson et al., 2013; Al-Ahmad et al., 2014; Ran et al., 2015).

Several virulence factors have been found to render specific *E. faecalis* strains more apt to cause disease or worsen disease symptoms. Enterococcal surface protein (*esp*) has been found to further adherence and colonization of cells and abiotic surfaces (Toledo-Arana et al., 2001; Paganelli et al., 2012). Gelatinase (*gelE*) is an extracellular metalloprotease, able to hydrolyze gelatin, collagen and hemoglobin, which has also been reported to contribute to bacterial adherence and biofilm formation (Franz et al., 2003; Kayaoglu and Orstavik, 2004). Aggregation substance (AS) has also been reported to increase adherence and invasion of eukaryotic cells (Kreft et al., 1992; Olmsted et al., 1994; Sussmuth et al., 2000) as well as promote biofilm formation (Chuang-Smith et al., 2010). Hyaluronidase (*hyl*), has been associated with virulence of enterococci in host tissue invasion (Kayaoglu and Orstavik, 2004; Fisher and Phillips, 2009). Furthermore, *E. faecalis* endocarditis antigen A (e*faA*) has been presumed to contribute to the adhesion of *E. faecalis* to heart cells in endocarditis (Singh et al., 1998; Reynaud af Geijersstam et al., 2007). Finally, cytolysin (*cyl*, beta-hemolysin) is a potent bacteriocin that exacerbates enterococcal infections in humans (Van Tyne et al., 2013). It is capable of lysing many prokaryotic cells, as well as erythrocytes and other eukaryotic cells (Van Tyne and Gilmore, 2014).

Beyond the mere possession of various virulence and resistance genes, *E. faecalis* is highly proficient in the exchange and passing on of many of these genes through horizontal gene transfer (Manson et al., 2010; Paganelli et al., 2012). In the last decade the transfer of antibiotic-resistance genes between different strains of *E. faecium*, as well as the transfer of vancomycin-resistance from *E. faecalis* to *Staphylococcus aureus* have been reported (Willems et al., 2001; Lester et al., 2006; Palmer et al., 2010).

Their frequent incidence in food and livestock could allow for a zoonotic route of transmission of *E. faecalis* to humans (Larsen et al., 2011; Hammerum, 2012). Transmission of *E. faecalis* of porcine origin or from raw milk to the human gastrointestinal tract through food has been suggested by Larsen et al. (2010) and Gelsomino et al. (2002).

Our group demonstrated that after the consumption of cheese, food-borne enterococci can integrate into the oral biofilm *in vivo* (Al-Ahmad et al., 2010), and recently Thurnheer and Belibasakis (2015) confirmed that *E. faecalis* is able to colonize an *in vitro* established six-species oral biofilm in high numbers. The finding that *E. faecalis* from food can incorporate into the oral biofilm and is prevalent in dental diseases raises the question as to whether the oral cavity serves as a reservoir for virulent and resistant strains of *E. faecalis* (Al-Ahmad et al., 2009, 2010).

For this reason, our study focussed on the presence of virulence traits in *E. faecalis* isolates from oral, clinical and food isolates. An additional aim was to determine the correlation of prevalent virulence genes with the ability to form biofilm. To accomplish this, characterization of virulence genes was performed by polymerase chain reaction (PCR) and phenotypic assays, and was then related to biofilm production. Additionally, phenotypic antibiotic resistance was tested and potential clonal relationships were assessed using pulsed-field gel electrophoresis (PFGE).

To our knowledge, this is the first study to combine data on virulence genes, antibiotic resistance, biofilm production and PFGE typing of *E. faecalis* isolates from the oral cavity, i.e., plaque/saliva and endodontic isolates, with other origins so as to improve our understanding of the oral cavity as a potential reservoir in the spread of virulent strains and the implications for endodontic and periodontitis-related treatment protocols.

## Materials and methods

### Bacterial isolates

A total of 97 *E. faecalis* isolates from four different sources were used for the experiments. Table 1 describes the sources of all isolates in detail. The oral isolates (endodontic infections, saliva and supragingival plaque) were collected from 2011 to 2014 in the Department of Operative Dentistry and Periodontology (University Freiburg Medical Center, Freiburg Germany), the clinical isolates were obtained from patients of the University Freiburg Medical Center in 2013 from the Department of Medical Microbiology and Hygiene (University Freiburg, Medical Center, Freiburg, Germany) and the raw milk isolates were received from the Bavarian Health and Food Safety Authority (Oberschleißheim, Germany) from 2014. The clinical and endodontic samples were gained after approval by the ethical committee of the Albert-Ludwigs-University Freiburg (Nr. 140/09, University of Freiburg, Germany). Standard protocols of the American Association of Endodontists as described in Schirrmeister et al. (2007) were followed to obtain endodontic samples. The species identity of the isolates was confirmed by amplification of a 16S rDNA fragment specific for *E. faecalis* (Table 2).

**Table 1:** *****Enterococcus faecalis*** isolates used in this study**.

**Number of isolates**	**Source**	**Characteristics**
30	Endodontic infection	Filled root canal
37	Dental plaque and saliva	
15	Nosocomial infection	9 Urinary tract infections 1 Wound (groin) 1 Intraoperative swab 1 Drainage secretion 1 Intraabdominal aspirate
		1 Blood culture
		1 Central venous catheter
15	Food	Raw milk

**Table 2:** **Primers used for the detection of different virulence genes of ***E. faecalis*** by PCR**.

**Target**	**Primer**	**Primer sequence (5′-3′)**	**Amplicon size [bp]**	**References**
*E. faecalis*	Efaec-F Efaec-R	GTTTATGCCGCATGGCATAAGAG CCGTACGGGGACGTTCAG	310	Siqueira and Rocas, 2004
*asa1*	ASA 11-F ASA 12-R	GCACGCTATTACGAACTATGA TAAGAAAGAACATCACCACGA	375	Vankerckhoven et al., 2004
*gelE*	GEL 11-F GEL 12-R	TATGACAATGCTTTTTGGGAT AGATGCACCCGAAATAATATA	213	Vankerckhoven et al., 2004
*cylA*	CYT I-F CYT IIb-F	ACTCGGGGATTGATAGGC GCTGCTAAAGCTGCGCTT	688	Vankerckhoven et al., 2004
*esp*	ESP 14F ESP 12R	AGATTTCATCTTTGATTCTTGG AATTGATTCTTTAGCATCTGG	510	Vankerckhoven et al., 2004
*hyl*	HYL n1-F HYL n2-R	ACAGAAGAGCTGCAGGAAATG GACTGACGTCCAAGTTTCCAA	276	Vankerckhoven et al., 2004
*efaA*	efaA-fw efaA-re	GCCAATTGGGACAGACCCTC CGCCTTCTGTTCCTTCTTTGGC	688	Creti et al., 2004

*The cycling conditions started with an initial denaturation at 94°C for 5 min, followed by 35 cycles with denaturation at 94°C for 1 min, annealing at 56°C for 1 min and extension at 72°C for 1 min, ending with a final extension at 72°C for 10 min*.

As a reference strain, *E. faecalis* 12030 was used, containing the genes for *gelE, cyl, efaA, esp*, and *asa1* and *E. faecium* 137 containing genes for *gelE, hyl*, and *esp*. Both strains were kindly provided by Prof. Dr. J. Hübner, Department of Medical Infectiology, Medical Center, University of Freiburg, Germany.

### Isolation of DNA

The DNA from all isolates was extracted and purified using DNeasy Blood and Tissue Kit (Qiagen, Hilden, Germany) according to the manufacturer's protocol for Gram-positive bacteria. The microbial DNA was eluted with 200 μl AE buffer (Qiagen) and stored at −20°C.

### PCR for the detection of *E. faecalis* virulence genes

Extracted DNA served as a template for the amplification of virulence genes and a 16S rDNA fragment specific for *E. faecalis*. All primer sequences and corresponding references are listed in Table 2. PCR amplification was performed in a total volume of 25 μl, containing 1 × PCR buffer (Qiagen), 0.2 mM each of the four deoxyribonucleoside triphosphates (dNTPs; PEQLAB, Erlangen, Germany), 0.5 mM of each of the primers, 2.5 U Taq-Polymerase (Qiagen) and 1 μl template DNA. The cycling conditions started with an initial denaturation at 94°C for 5 min, followed by 35 cycles with denaturation at 94°C for 1 min, annealing at 56°C for 1 min and extension at 72°C for 1 min, ending with a final extension at 72°C for 10 min.

Each set of PCR reactions included a no-template control and a positive control. The amplification products were analyzed by gel electrophoresis using a 1.0% agarose gel.

### Phenotypic assays for production of gelatinase and hemolysin

The strains were tested for the production of gelatinase using stab cultures containing 12% gelatin. The inoculum was placed in the center of the tubes and incubated at 37°C for 24 h and then kept at 4°C for 4 h. Positive gelatinase production was evident as liquefied agar.

Hemolytic activity was analyzed by streaking a colony on Mueller-Hinton Agar (Becton Dickinson, Heidelberg, Germany) containing 5% horse blood. After incubation at 37°C for 24 h, a clear halo around the colonies indicated hemolysin production (Coque et al., 1995).

### Antibiotic susceptibility testing

The susceptibilities of the isolates to the clinically relevant antibiotics, Penicillin G, ampicillin, ampicillin + sulbactam, imipenem, ciprofloxacin, levofloxacin, teicoplanin, vancomycin, tigecyclin, cotrimoxazol, linezolid, nitrofurantoin, high-level gentamicin, erythromycin, tetracyclin, and trimethoprim/sulfamethoxazol were tested using the VITEK AST-P616 card (bioMérieux Deutschland GmbH, Nürtingen, Germany). Testing was performed according to the manufacturer's instructions and interpreted as indicating susceptible, intermediate, or resistant categories according to the EUCAST standards (The European Committee on Antimicrobial Susceptibility Testing) as implemented in the VITEK system.

### Biofilm production

The capacity for biofilm production of the *E. faecalis* isolates was assessed with a biofilm plate assay as previously described (Al-Ahmad et al., 2014). In brief, the isolates were cultivated in 5 ml tryptic soy broth (Oxoid, Wesel, Germany) at 37°C overnight. Then the bacterial count of the cultures was determined to be in the range of 10^8^ cfu/ml. The wells of Polystyrene 96-well plates (Greiner, Bio-one, Frickenhausen, Germany) were filled with 180 μl fresh Tryptic Soy Broth (Oxoid), and 20 μl of overnight culture were pipetted into each. After incubation of the plates for 48 h at 37°C, the culture medium was discarded and the plates were washed three times with 300 μl phosphate buffered saline (PBS, Sigma-Aldrich Chemie GmbH) to remove non-adherent bacterial cells. The plates were then air dried and stained with 0.1% crystal violet solution (Median Diagnostics GmbH, Dunningen, Switzerland) for 10 min. To remove excess stain, the plates were washed three times with 200 μl distilled water. After drying the plates at 60°C for 10 min, 50 μl alcohol (99.9%, absolute for analysis; Merck, Darmstadt, Germany) were added to each well to resolubilize the dye. A Tecan Infinite 200 plate reader (Tecan Austria GmbH, Grödig, Austria) was used to measure the optical density of each well at 595 nm. All measurements were performed in quadruplicates, the experiments were repeated twice and the mean values of all measurements were calculated. Three different categories for the level of biofilm-forming capacity were determined according to two different cut-off values: C1 = biofilm non-producer, C2 = moderate biofilm producer, C3 = high biofilm producer. The low cut-off value was determined by adding three standard deviations of the blank to the negative control and the high cut-off was defined as three times the low cut-off value.

### Genotyping by pulsed-field gel electrophoresis (PFGE)

PFGE was used to compare *E. faecalis* isolates of different origins and was performed as previously described (Al-Ahmad et al., 2010). In brief, prepared sample plugs were digested with 20 U SmaI (New England Biolabs, Beverly, MA) and subsequently loaded on a 1% agarose gel (LE GP agarose; Biozym, Hess, Oldendorf, Germany) in 0.5% Tris-Borate-EDTA buffer (TBE, Sigma-Aldrich Chemie GmbH). PFGE was performed using a CHEF-DR II apparatus (Bio-Rad Laboratories, Munich, Germany) with the following parameters: 1–11 s for 13 h and 11–30 s for 13 h at 6 V/cm and 14°C. The gel was stained with ethidium bromide to visualize DNA bands. All PFGE patterns were analyzed using the BioNumerics software (Applied Maths) with the control strain *Staphylococcus aureus* NCTC 8325 as a molecular size standard for normalization. The fingerprints were compared using the Dice coefficient (with 1% tolerance and 0.5% optimization) and cluster analysis with the unweighted pair group method using arithmetic averages (UPGMA). Strains were considered to be possibly related if their patterns differed by one to three bands (Tenover et al., 1995).

### Statistical evaluation

The correlation between biofilm formation capacity (biofilm non-producer, moderate biofilm producer, high biofilm producer), the origin of the isolates and the presence of different virulence genes was determined by the Institute for Medical Biometry and Statistics, Center for Medical Biometry and Medical Informatics, Freiburg, Germany, using Fishers' exact test. Ordinal logistic regression was used to check for an influence of the different virulence genes on biofilm formation capacity. The method of Scheffe was applied to correct for the multiple testing problem and allowed for the adjustment of *p*-values with 95% confidence intervals (CI).

## Results

A total of 97 *E. faecalis* isolates from oral, food and clinical samples were analyzed for the presence of several virulence genes, the phenotypic expression of two virulence genes and their ability to form biofilm. A number of isolates was tested for possible clonal relations.

### Presence of virulence genes in oral, food, and clinical isolates

The presence of genes that encode aggregation substance (*asa1*), gelatinase (*gelE*), cytolysin (*cylA*), hyaluronidase (*hyl*), endocarditis antigen A (*efaA*) and enterococcal surface protein (*esp*) was detected by PCR. Regardless of their origin, all tested isolates possessed the *efaA* gene, whereas none of the isolates possessed the gene for hyaluronidase (*hyl*). Concerning the other virulence genes, 99% of all isolates were positive for *gelE*, 92% for *asa1*, 70.5% for *esp* and 47.4% for *cylA*.

**Figure 1:**
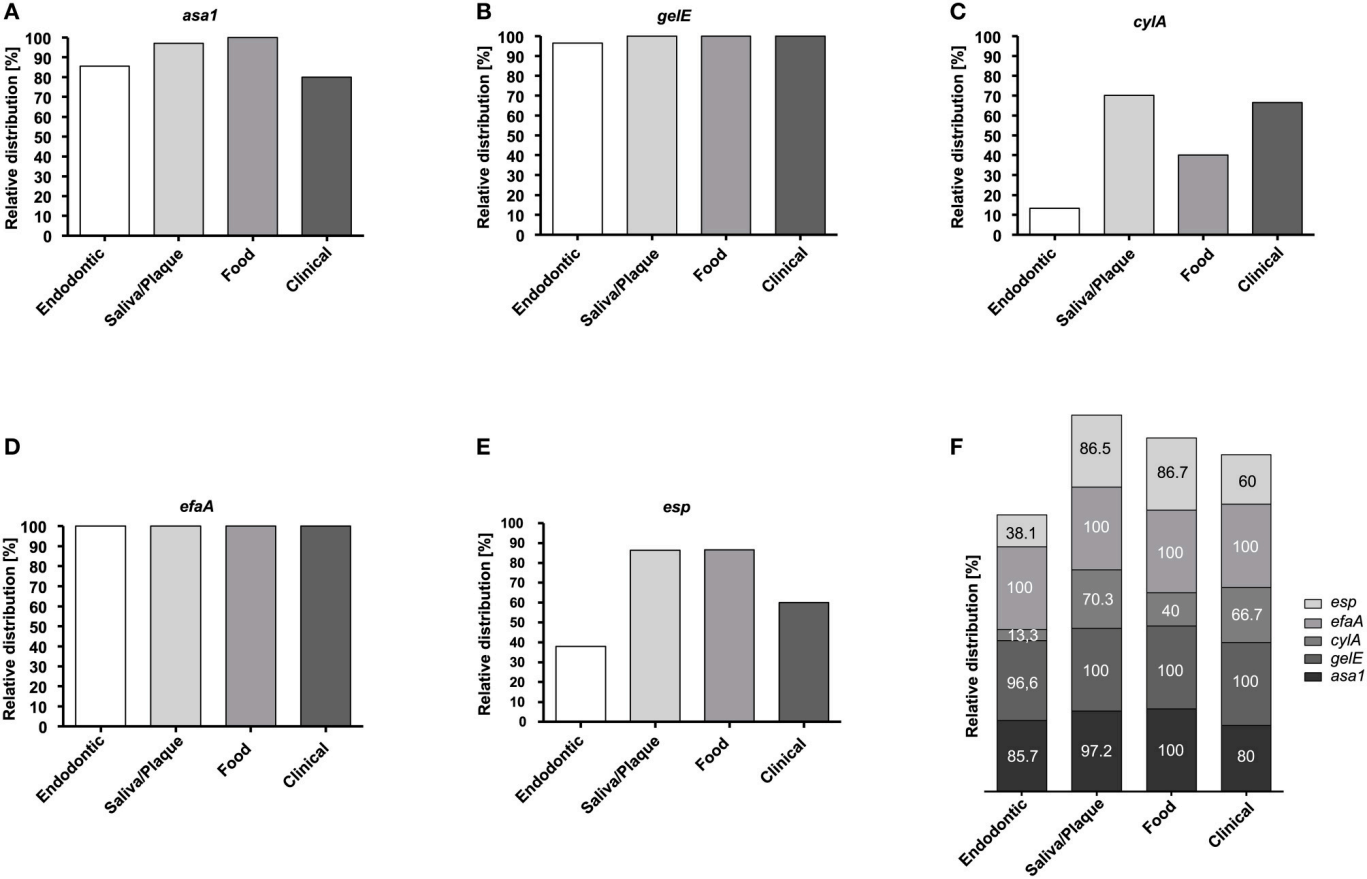
**Relative distribution of virulence genes in ***E. faecalis*** isolates according to their origin**. Relative distribution of *asa1* **(A)**, *gelE* **(B)**, *cylA* **(C)**, *efaA* **(D)**, and *esp* **(E)** in endodontic (*n* = 21), plaque/saliva (*n* = 37), food (*n* = 15), and clinical (*n* = 15) isolates. Overall relative distribution of *asa1, gelE, cylA, efaA*, and *esp* **(F)** in *E. faecalis* isolates from four different origins.

**Figure 2:**
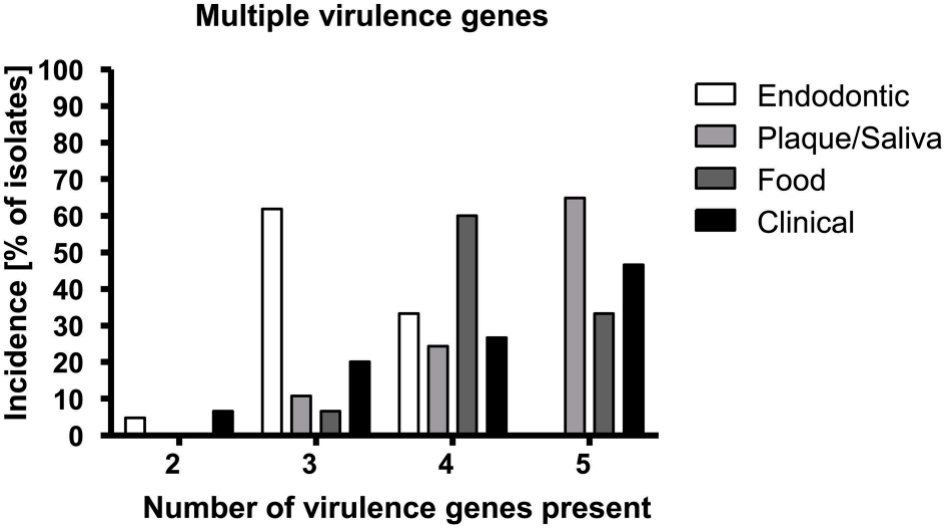
*****E. faecalis*** isolates from different origins show different incidences for multiple virulence genes**. The x-axis displays the number of detected virulence genes; the y-axis shows the percentage of isolates of the respective origin possessing the respective number of virulence genes.

Figures 1A–F depicts the percentage of each virulence gene in the samples according to their origin while Table 3 shows all of the detected virulence genes from the different isolates in detail. Endodontic isolates possessed the fewest virulence genes compared to the isolates from other sources, while plaque/saliva isolates possessed the highest percentage of all the detected virulence genes compared to all other groups (Figure 1F). The incidence of multiple virulence genes present at the same time was high in clinical (73.3%), food (93.3%), and plaque/saliva (89.2%) isolates (Figure 2). Food isolates, plaque/saliva isolates and clinical isolates contained 4–5 different virulence genes at the same time.

**Table 3:** **Origin, presence of ***asa1, gelE, cylA, hyl, efaA***, and ***esp*** genes, production of gelatinase and hemolysin and ability to form biofilm of ***E. faecalis*** isolates from four different sources**.

**Isolate**	***asa1***	***gelE***	**Gelatinase production^a^**	***cylA***	**Hemolysin production^b^**	***hyl***	***efaA***	***esp***	**Biofilm formation^c^**
**ENDODONTIC**									
1aR1	−	+	−	−	−	−	+	+	++
1anR8	+	+	−	−	−	−	+	−	++
11aRSP	+	+	−	−	−	−	+	+	+
12aSP	+	+	−	−	−	−	+	+	+
17aSP	+	+	−	−	−	−	+	−	+
21aSP	−	−	−	−	−	−	+	+	++
33aR8	+	+	−	−	−	−	+	−	+
39aR6SP	+	+	−	−	−	−	+	−	n.d.
44aR6	−	+	−	−	−	−	+	−	+
44aREnA	+	+	−	−	−	−	+	−	+
44aF6	+	+	−	−	−	−	+	−	+
44aFEnA	+	+	−	−	−	−	+	−	+
44anR7	+	+	−	−	−	−	+	−	−
44anR10	+	+	−	−	−	−	+	−	−
44anF7	+	+	−	−	−	−	+	−	−
45aSP7	+	+	−	−	−	−	+	−	+
52F7	n.d.	+	−	−	−	n.d.	n.d.	n.d.	n.d.
52R7	n.d.	+	−	−	−	n.d.	n.d.	n.d.	n.d.
53F6	n.d.	+	−	+	−	n.d.	n.d.	n.d.	n.d.
53R6	n.d.	+	−	−	−	n.d.	n.d.	n.d.	n.d.
54F11	n.d.	+	−	+	+	n.d.	n.d.	n.d.	n.d.
54QC6	n.d.	+	−	+	+	n.d.	n.d.	n.d.	n.d.
55F7	n.d.	+	−	−	−	n.d.	n.d.	n.d.	n.d.
55R6	n.d.	+	−	+	−	n.d.	n.d.	n.d.	n.d.
57R9	n.d.	+	+	−	−	n.d.	n.d.	n.d.	n.d.
RGFR−81G8	+	+	−	−	−	−	+	+	n.d.
RG20R72C3	+	+	−	−	−	−	+	−	+
RG18F102F2	+	+	−	−	−	−	+	+	−
MFCT7501C6	+	+	−	−	−	−	+	+	−
MFCT23501A1	+	+	−	−	−	−	+	+	+
**PLAQUE/SALIVA**									
90	+	+	−	−	−	−	+	−	−
91	+	+	+	−	−	−	+	−	+
223	+	+	−	−	−	−	+	−	+
254	+	+	−	+	+	−	+	+	+
255	+	+	+	−	−	−	+	+	+
281	+	+	−	+	−	−	+	+	+
282	+	+	−	+	+	−	+	+	−
288	+	+	−	+	+	−	+	+	−
289	+	+	−	+	+	−	+	+	−
290	+	+	−	+	+	−	+	+	+
291	+	+	−	+	+	−	+	+	+
292	+	+	−	+	+	−	+	+	+
293	+	+	−	+	+	−	+	+	+
294	+	+	−	+	+	−	+	+	+
295	+	+	−	+	+	−	+	+	+
296	+	+	−	+	+	−	+	+	+
319	+	+	−	+	+	−	+	+	−
327	+	+	−	+	+	−	+	+	+
351	+	+	+	−	−	−	+	+	+
352	+	+	−	−	−	−	+	+	−
353	+	+	−	−	−	−	+	+	+
354	+	+	−	+	−	−	+	+	+
357	+	+	−	+	−	−	+	−	++
358	+	+	−	−	−	−	+	+	+
359	+	+	+	+	−	−	+	+	+
360	−	+	−	−	−	−	+	+	−
361	+	+	+	−	−	−	+	+	+
383	+	+	−	−	−	−	+	+	+
446	+	+	−	+	+	−	+	+	−
447	+	+	−	+	+	−	+	+	+
448	+	+	−	+	+	−	+	+	−
449	+	+	−	+	+	−	+	+	−
450	+	+	−	+	+	−	+	+	+
451	+	+	−	+	+	−	+	+	+
452	+	+	−	+	+	−	+	+	−
478	+	+	−	+	−	−	+	−	+
513	+	+	−	+	−	−	+	+	++
**FOOD**									
F2/19	+	+	−	−	−	−	+	+	−
E392	+	+	−	+	−	−	+	+	−
C339	+	+	−	−	−	−	+	+	+
C350	+	+	−	+	+	−	+	+	−
C409	+	+	−	−	−	−	+	−	++
C528	+	+	−	+	+	−	+	−	+
C671	+	+	−	−	−	−	+	+	−
C686	+	+	−	−	−	−	+	+	+
C725/3	+	+	+	+	−	−	+	+	−
C729	+	+	−	+	−	−	+	+	+
C737/1	+	+	−	−	−	−	+	+	−
C739	+	+	+	−	−	−	+	+	++
C771	+	+	−	−	−	−	+	+	−
C890	+	+	−	−	−	−	+	+	−
C906/1	+	+	−	+	−	−	+	+	−
**CLINICAL**									
110028	+	+	−	−	−	−	+	−	−
110035	+	+	−	+	+	−	+	+	+
110047	+	+	+	−	−	−	+	−	+
110053	+	+	−	+	−	−	+	+	+
109891	+	+	+	+	−	−	+	−	++
109898	+	+	−	+	+	−	+	+	+
229355	−	+	−	+	+	−	+	−	+
512106	−	+	−	−	−	−	+	−	+
512118	+	+	−	+	+	−	+	+	−
512129	−	+	−	−	−	−	+	+	++
512176	+	+	−	+	+	−	+	+	−
512188	+	+	−	+	−	−	+	−	++
512276	+	+	−	+	+	−	+	+	+
512298	+	+	−	−	−	−	+	+	−
512359	+	+	−	+	+	−	+	+	+
Total samples [%]	92.0	98.9	10.3	47.4	65.96	0	100	70.5	10/86^++^ = 11.63 47/86+ = 54.65 29/86^−^ = 33.72

a and b *Phenotypic assays*.

c *^−^Biofilm non-producer, ^+^moderate biofilm producer, ^++^high biofilm producer*.

### Phenotypic assays for gelatinase and hemolysin production

Silent gelatinase genes (*gelE*) were present in isolates of all sources. Gelatinase activity occurred in 13% of the plaque/saliva, food and clinical isolates but in only 3.3% of the endodontic isolates. Hemolytic activity occurred in 54.1% of the plaque/saliva isolates, 46.6% of the clinical isolates, 13.3% of the food isolates and only 6.6% of the endodontic isolates.

### Antibiotic susceptibility testing

The detailed results presenting the MIC values for the tested isolates are shown in Supplementary Table S1. Isolates that were considered resistant against an antibiotic according to the EUCAST standards were marked in bold, intermediate results in bold italics. All the isolates from the different origins were resistant against erythromycin but susceptible to ampicillin, ampicillin-sulbactam, imipenem, tigecycline, nitrofurantoin and for the glycopeptides teicoplanin as well as vancomycin. For trimethoprim/sulfamethoxazol most isolates showed an intermediate result (MIC ≤ 10.0 mg/L), 2 food isolates (2/14) and 7 clinical isolates (7/15) were resistant against it (MIC 80− ≥ 320 mg/L). Tetracycline-resistance was wide-spread and was revealed for 10 endodontic isolates (10/28) and for almost all plaque/saliva (31/36), food (13/14) and clinical isolates (13/15), with all the isolates showing MIC values ≥16 mg/L. High-level gentamicin synergy resistance was shown for many plaque/saliva (19/36) and clinical isolates (8/15) but was not found for either endodontic or food isolates. In addition, several clinical isolates (5/15) presented MIC values showing resistance against levofloxacin and ciprofloxacin (≥8 mg/L each), and one endodontic isolate showed intermediate results for ciprofloxacin. Linezolid resistance was found for one other endodontic isolate.

### Biofilm formation capacity

**Figure 3:**
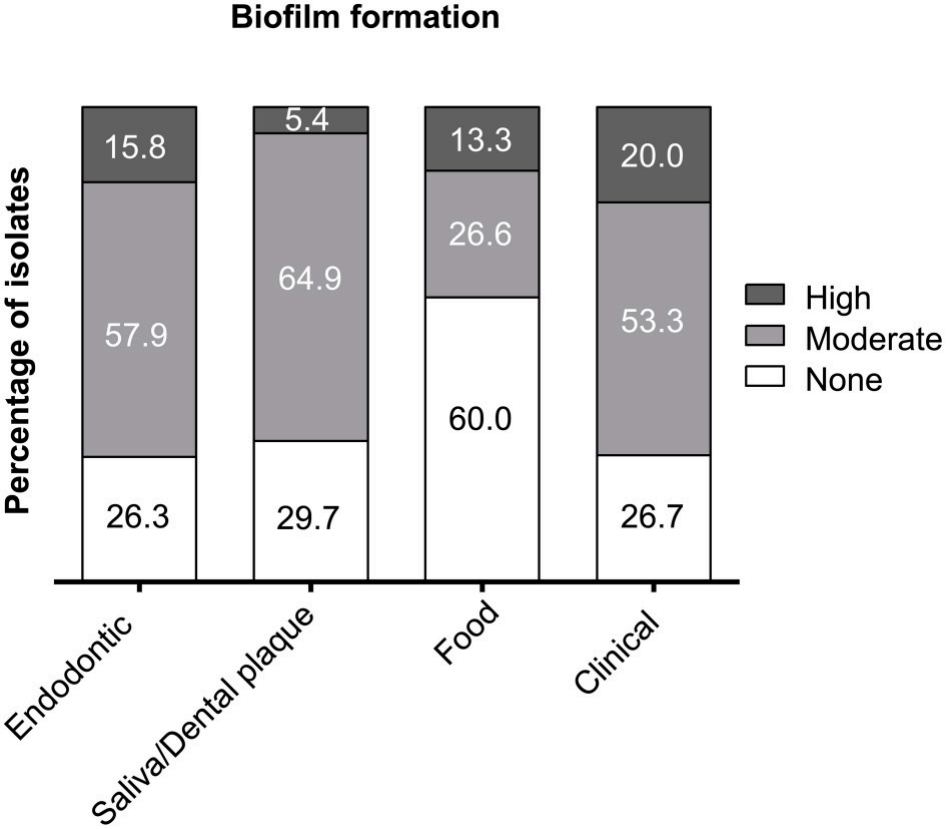
**Capacity for biofilm formation of ***E. faecalis*** isolates from endodontic, plaque/saliva, food, and clinical samples**. The x-axis shows the origin of the isolates and the y-axis displays the percentage of non-producers, moderate and high biofilm producers.

**Figure 4:**
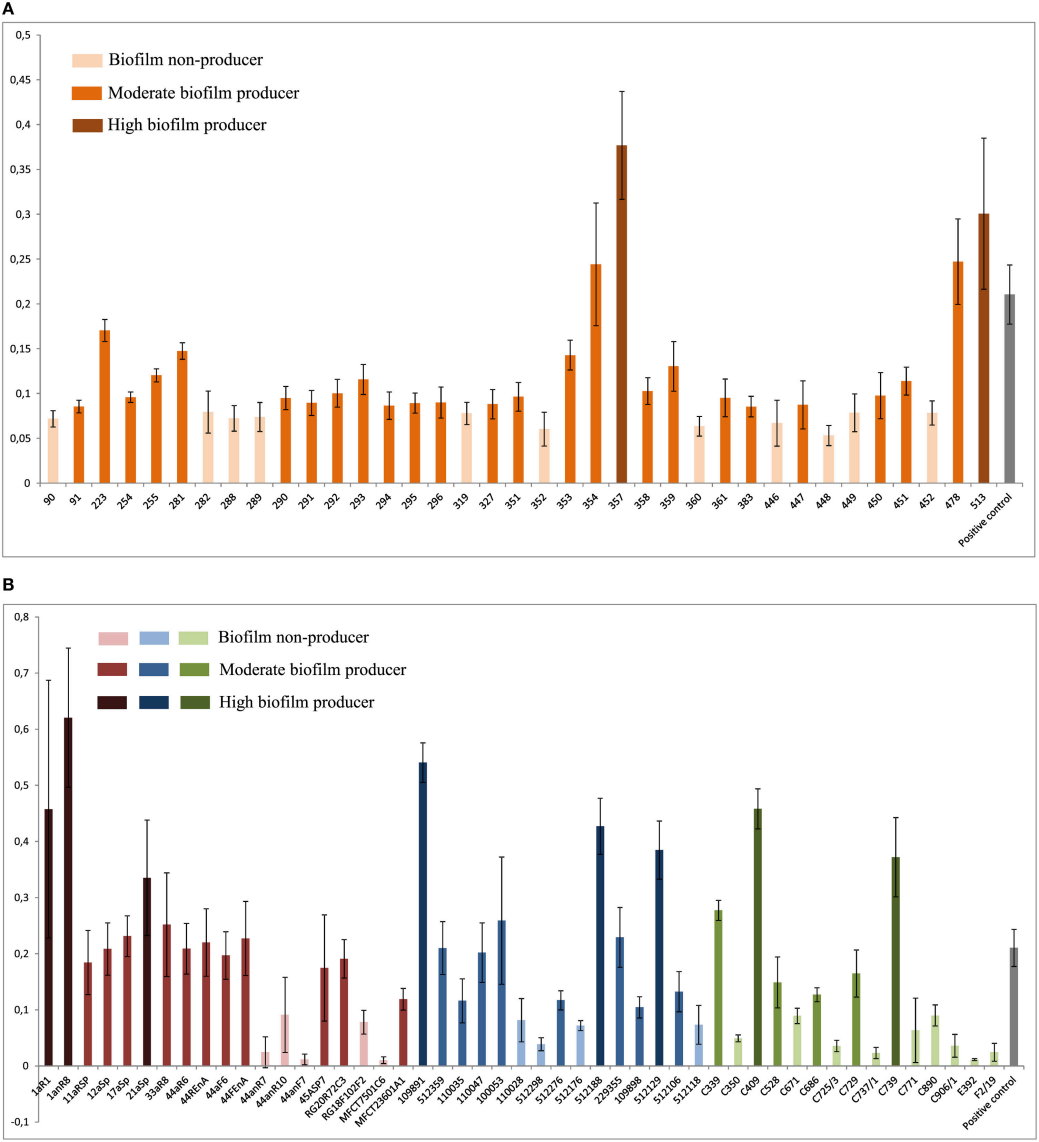
**Results of the biofilm plate assay**. Means and standard deviations of four repeated measurements are presented. X-axis shows isolates; y-axis shows OD_595_ values. **(A)** Biofilm formation by plaque/saliva isolates; low cut-off: 0.0836; high cut-off: 0.2506. **(B)** Biofilm formation by endodontic, clinical, and food isolates; low cut-off: 0.0992; high cut-off: 0.2976. Red: Endodontic isolates; blue: Clinical isolates; green: Food isolates.

The food isolates presented the lowest capacity for biofilm formation (Figure 3) with 60% non-biofilm producers. The majority of the endodontic isolates (73.7%) were able to form biofilm (57.9% moderate producers, 15.8% high producers). Most clinical isolates had moderate or high biofilm formation capacity (73.3%), displaying the highest percentage of high biofilm producing isolates (20%) among the tested isolates. Also most plaque/saliva isolates proved to be biofilm producers (64.9 moderate, 5.4% high). Altogether, 54.7% of the tested isolates had moderate biofilm forming abilities, 11.6% were high biofilm producers and 33.7% did not produce biofilm at all. The OD-values for all the isolates are shown in Figure 4 and the capacity for biofilm formation for the individual isolates are listed in Table 3.

### Correlation of isolate origin, presence of virulence genes, and biofilm formation capacity

Statistical evaluation of possible correlations between the respective origins of the isolates with the presence of certain virulence genes revealed that the presence of *cylA* correlated significantly with the origin of the isolates. Of the cylA-positive isolates, 56.5% originated from plaque/saliva samples, whereas 51.0% of the *cylA*-negative isolates stemmed from endodontic samples (*p* < 0.001). Also the presence of *esp* correlated significantly with the origin of the isolates (*p* < 0.001), 51.6% of the *esp*-positive isolates originated from plaque/saliva samples, whereas 50% of the *esp*-negative samples were recovered from endodontic samples.

As far as their ability to form biofilm is concerned, statistical evaluation showed a tendency of *asa1* positive isolates to correlate with moderate biofilm formation capacity (*p* = 0.058), with 55.7% of all *asa1*-possessing isolates showing moderate biofilm production.

### Analysis of isolates with PFGE

**Figure 5:**
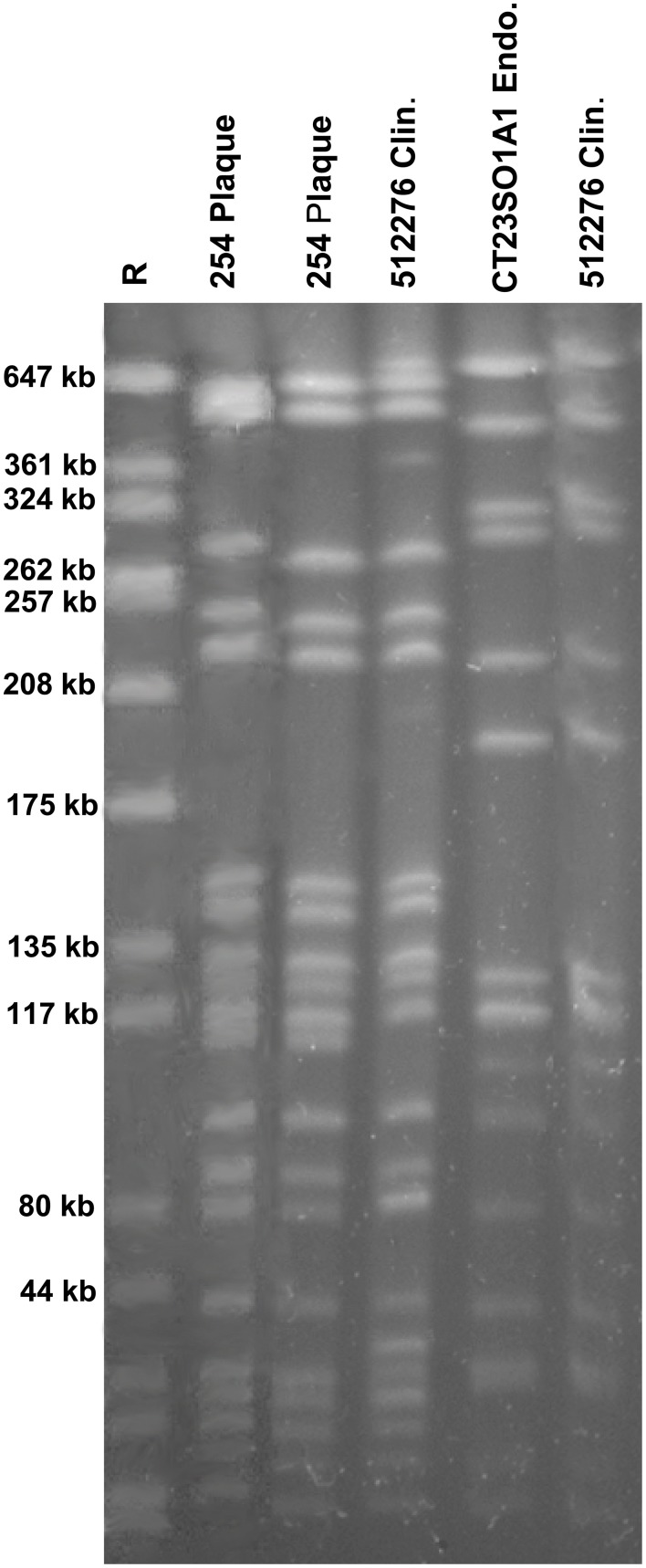
**PFGE patterns from ***E. faecalis*** isolates from four samples, each of different origin (plaque, clinical, endodontic, food)**. The plaque isolate (254) as well as the clinical isolate (512276) belonged to the same subtype, and the endodontic isolate (CT23SO1A1) and the food isolate (C906/1) were indistinguishable.

One endodontic isolate (MFCT23501A1) and one food isolate (C906/1) seemed to be closely related, belonging to the same PFGE subtype. These two isolates correspond with regard to their virulence gene pattern (*gelE, efaA, esp, asa1*), except that the food isolate was additionally positive for *cylA*. Furthermore, one plaque isolate (254) and one clinical isolate (512276, from an UTI) belonged to the same PFGE subtype and also revealed identical virulence gene patterns (*gelE, cylA, efaA, esp, asa1*), both showing hemolytic activity and both possessing moderate biofilm formation capacity. The corresponding PFGE analysis is shown in Figure 5. For all other samples no clustering was shown for any isolates from the different sources, the corresponding results are reported in the supplementary information (Supplementary Figures S1A–F).

## Discussion

*E. faecalis* strains can cause severe nosocomial infections and are detected as food contaminants and used as starter cultures or probiotics. They are associated with different dental diseases and can integrate into the oral biofilm. The role of *E. faecalis* in the oral cavity has not been clarified yet. Hence, this study compared the incidence of several virulence genes, phenotypic expression of gelatinase and hemolysin, antibiotic susceptibility and the ability to produce biofilm in clinical, food, endodontic and plaque/saliva isolates.

The isolates show characteristic differences in their virulence gene patterns and their ability for biofilm formation, with all isolates possessing multiple virulence genes. Most notably, the clinical isolates from various infections and the plaque/saliva isolates derived from healthy individuals possessed the highest percentage of virulence genes, the highest accumulation of multiple virulence genes and simultaneously a high ability to form biofilm. The plaque/saliva isolates even exceeded the clinical isolates regarding the incidence of *esp* and *asa1*. There were no differences found between the different origins in the abundance of *efaA* (100%) and *hylA* (0%) and almost all isolates possessed *gelE* (96.7–100%), yet a high proportion of this gene was silent. The biofilm formation capacity of clinical and oral isolates was considerably higher than that of food isolates which still presented a high abundance of virulence genes.

The prevalence of *E. faecalis* in the oral cavity has been investigated and reported to be comparatively low in healthy individuals, i.e., 1–20% (Sedgley et al., 2004, 2006) yet elevated up to 68% in patients with dental diseases i.e., periodontitis, caries and endodontic infections (Sedgley et al., 2006; Souto and Colombo, 2008; Kouidhi et al., 2011). This led to the assumption that *E. faecalis* is not a member of the oral microbiota. Consequently, supragingival plaque from healthy individuals has not been analyzed with regard to *E. faecalis* and its virulence and biofilm traits. Therefore, the high incidence of *asa1, gelE, efaA, esp*, and *cylA* is a novel finding and stresses that the healthy oral cavity has been unjustifiably neglected. In subgingival plaque samples of chronic periodontitis, *asa1, gelE, cylA*, and *esp* were detected in 58, 79, 38, and 50% of the isolates (Sun et al., 2012), whereas in our study, these genes were found in 97.2, 100, 70, and 86.5% of the isolates from plaque/saliva, respectively. Creti et al. (2004) analyzed these virulence genes in commensal strains from feces and throats and also found *asa1, gelE, cylA*, and *esp* in lower abundances, i.e., in 90, 40, 30, and 20% of the isolates. However, the percentages of *gelE* and *cylA* showing phenotypic activity were higher in the study of Creti et al. (2004) compared to our results.

The clinical samples in our study also revealed higher abundances of *asa1, gelE, cylA*, and *esp* than those reported by Creti et al. (2004) or Eaton and Gasson (2001). This might be due to the sources of our clinical isolates, since a high proportion of our clinical samples were derived from urinary tract infections, which have been reported to harbor a higher percentage of these traits (Giridhara Upadhyaya et al., 2010; Tsikrikonis et al., 2012; Kafil et al., 2013; Sharifi et al., 2013).

Several authors have compared isolates from food and clinical samples and found the tendency that clinical isolates possessed more virulence genes than food strains, and that these in turn had more virulence genes than strains from starter cultures (Eaton and Gasson, 2001; Semedo et al., 2003; Medeiros et al., 2014). In contrast, in food isolates of our study, *asa1* and *esp* were more abundant than in clinical isolates. Our food isolates also possessed more *esp* and *gelE* than the food isolates studied by Eaton and Gasson (2001). A possible explanation could be that all our food isolates originated from raw milk, an evident restriction of this study. Still, the high percentage of virulence genes in these isolates is noteworthy and in agreement with Moraes et al. (2012) who tested raw milk and cheese and found high abundances of *asa1, gelE*, and *efaA*, as well as high percentages of hemolytic activity.

The endodontic isolates analyzed in this study revealed a lower abundance of *cylA* and *esp* than all the other isolates. A high incidence for *gelE, efaA*, and *asa1* with low abundances for *cylA* and *esp* in endodontic samples from treated root canals have also been found by several authors (Sedgley et al., 2005b; Reynaud af Geijersstam et al., 2007; Zoletti et al., 2011). Phenotypic gelatinase activity has been reported in 47–70% of positive isolates (Sedgley et al., 2005b; Reynaud af Geijersstam et al., 2007; Zoletti et al., 2011) which is in contrast to our study revealing gelatinase activity in only 3.3% of isolates. On the other hand, the low hemolytic activity found by Sedgley et al. (2005b) and Reynaud af Geijersstam et al. (2007) is concurrent with our results of 6.6% hemolytic activity in endodontic isolates.

The *gelE* virulence gene seems widespread in isolates from different sources, yet often silent. This is a consistent finding in various studies (Eaton and Gasson, 2001; Creti et al., 2004; Sedgley et al., 2005a; Dahlen et al., 2012) and is ascribed to the difference between *in vivo* and *in vitro* conditions, or the absence of other genes that are required for expression (Sedgley et al., 2005a; Seputiene et al., 2012). Phenotypic expression of gelatinase in our study occurred in only a low percentage of the isolates of any source and the elevated gelatinase activity in clinical samples found by Coque et al. (1995) was not observed in this study. Hemolytic activity varied for the isolates in this study, being higher for plaque/saliva and clinical isolates, which also showed a higher abundance of the *cylA* genotype, and lower for endodontic and food isolates. Similarly to Buhnik-Rosenblau et al. (2013), who found higher hemolytic activity in commensal strains than in clinical isolates, our plaque/saliva isolates showed even higher hemolytic activity (54.1%) than the clinical isolates (46.6%).

The resistance of oral *E. faecalis* isolates to tetracycline, erythromycin and high level gentamicin and of one endodontic isolate against ciprofloxacin and linezolid resp. must not be neglected. It is relevant regarding the treatment options for oral diseases, since tetracyclines and ciprofloxacin have been administered in periodontitis (Rams et al., 1992; Roberts and Mullany, 2010), and at the same time regarding the potential spread of these genes to other strains or species within the oral biofilm. In previous studies similar resistance patterns were found for endodontic and periodontal *E. faecalis* isolates (Rams et al., 1992; Al-Ahmad et al., 2014), yet the erythromycin resistance in our study is more prevalent. Overall, the clinical isolates showed resistance against more antibiotics than isolates from the other sources, still the high-level gentamicin synergy resistance for 52% of the plaque/saliva isolates is noteworthy and corresponds with the rate for the clinical isolates (53%) in our study.

The microtiter plate assay, used to assess the biofilm formation, has been shown to be a useful tool to compare the *in-vitro* biofilm formation capacity of isolates of different origins (Stepanovic et al., 2000; Al-Ahmad et al., 2014). However, although the measured OD values corresponded well with the actual number of cells present in the biofilm in a recent study of Leuck et al. (2014), the compared *ex-vivo* biofilm formation of clinical *E. faecalis* isolates in a heart valve tissue assay was found to be considerably higher than measured by the plate assay. This suggests that the *in-vivo* biofilm formation ability of the isolates in our study might be even higher than reported by the plate assay. Nevertheless, limiting ourselves to the comparison of the different origins tested in our study, there were distinct differences in the results of the plate assay.

The ability for moderate to high biofilm formation was present in the majority of the plaque/saliva isolates as well as clinical and endodontic isolates. Only food isolates showed a high percentage of non-biofilm producers and had less capacity for biofilm formation than all human-derived isolates. Other authors have reported higher biofilm formation in clinical samples than e.g., in environmental or animal samples (Tsikrikonis et al., 2012). This is in agreement with our results regarding food isolates. However, it has been reported for milk isolates and other animal-derived *E. faecalis* strains that biofilm production can depend on growth conditions (Baldassarri et al., 2001; Elhadidy and Elsayyad, 2013; Jahan and Holley, 2014). Concerning biofilm formation of clinical isolates vs. commensal human isolates, there are some contradicting studies. Tsikrikonis et al. (2012) and Duggan and Sedgley (2007) did not observe biofilm formation to be dependent on the clinical or commensal origin of the isolates, whereas Giridhara Upadhyaya et al. (2010) found higher biofilm production in clinical isolates than in commensal fecal strains. A study by Mohamed et al. (2004) showed that endocarditis isolates were associated with more biofilm formation than other isolates. Contrary to that study, Johansson and Rasmussen (2013) reported that commensal isolates from human feces formed even more biofilm than endocarditis isolates and concluded that biofilm formation might be a prerequisite for bacterial colonization of the gastrointestinal tract. Accordingly, the high percentage of biofilm producers in the plaque/saliva samples found in this study may indicate that this trait is essential for integration and permanence in the oral biofilm. The biofilm formation capacity of the endodontic samples (74% moderate and high biofilm producers) in this study is comparable with the results of Wang et al. (2011) who found 75% biofilm producers within endodontic samples. Our group tested endodontic isolates from different species and found two moderate biofilm producers among 5 of the *E. faecalis* isolates tested, with both isolates also showing antibiotic resistance (Al-Ahmad et al., 2014). Since infections of the oral cavity, e.g., periodontitis apicalis or periimplantitis, are caused by biofilms the present study reveals that *E. faecalis* strains of different sources could integrate into different compartments of the oral cavity, contributing to the infection process through biofilm formation.

A positive correlation between present virulence genes and the ability to form biofilm has been studied and confirmed by several authors. Tsikrikonis et al. (2012) has reported higher biofilm formation in *esp*-positive and gelatinase-positive isolates. Similarly, Toledo-Arana et al. (2001) and Soares et al. (2014) found significant associations of *esp* and *gelE* with the ability to form biofilm in isolates from clinical samples. In contrast, in our study a number of *esp*-negative isolates were moderate or high biofilm producers. This is also confirmed by Kristich et al. (2004), who reported that *esp* is not an essential determinant in biofilm formation. Mohamed and Murray (2005) found no significant correlation between the presence of *esp* and *gelE* and biofilm formation in a large collection of *E. faecalis* isolates, even though the optical density (OD) values for biofilm formation were highest for *esp*/*gelE* positive isolates and lowest for *esp*/*gelE*-negative isolates. Therefore, they concluded that other factors contribute to these differences in biofilm formation. Similarly, our study did not reveal any correlation between *esp*- and *gelE*-positive isolates and biofilm formation. Nevertheless, *asa1*-positive isolates showed a tendency to be associated with moderate biofilm production. Aggregation substance (*asa1*) as virulence gene has been reported to promote biofilm formation by Chuang-Smith et al. (2010) and also to increase adherence to and invasion of eukaryotic cells (Kreft et al., 1992; Olmsted et al., 1994; Sussmuth et al., 2000) in this way exacerbating the symptoms of infections. Soares et al. (2014) similarly observed an association of aggregation substance with biofilm formation.

Concerning possible clonal relationships between the different isolates, PFGE analysis revealed very high genetic heterogeneity, which is a common finding in studies comparing strains of diverse origins, periods of time and places of origin (Creti et al., 2004; Sedgley et al., 2004; Zoletti et al., 2011; Golinska et al., 2013). However, one plaque isolate and one clinical isolate belonged to the same subtype, suggesting a possible connection between the strains found in the oral cavity and infection-derived strains. Furthermore, one endodontic isolate and one food isolate were found to be closely related, also possessing the same virulence genes and biofilm formation capacity. A probable foodborne route of infection with *E. faecalis* has previously been proposed by Larsen et al. and Gelsomino et al. as well as Zehnder and Guggenheim (Gelsomino et al., 2002; Zehnder and Guggenheim, 2009; Larsen et al., 2010).

In conclusion, in the present study the isolates from all origins (food, clinical samples, endodontic infections, and plaque/saliva) possessed high percentages of the tested virulence genes, varying only with regard to *asa1, cylA*, and *esp*. Plaque/saliva isolates revealed both the highest percentages of virulence genes and of gelatinase/hemolytic activity as well as resistance against several antibiotics and simultaneously showed high biofilm formation capabilities. Therefore, the pathogenic potential of isolates from plaque/saliva seems to be as great as that of clinical isolates. Oral isolates from plaque and saliva are armed with the genetic equipment of these virulence factors, and due to the constant “coming and going” of bacterial strains in the oral cavity, *E faecalis* could potentially spread its virulence and antibiotic resistance genes to other species. In particular, taking into account the fact that horizontal gene transfer rates are increased for species found in biofilm communities compared to planktonic populations (Al-Ahmad et al., 2014), and that the integration of food-borne *E. faecalis* from cheese into the oral biofilm has been observed (Al-Ahmad et al., 2010), this could represent a possible scenario.

## Author contributions

ACA participated in the design of the study, analyzed and interpreted the data and drafted the manuscript. LK participated in the analysis of the data and revised the manuscript critically for important intellectual content. IH, NA, and AW participated in the acquisition of the samples and revised the manuscript critically for important intellectual content. DJ, JW, and EH revised the manuscript critically for important intellectual content. AA participated in the design of the study and revised the manuscript critically for important intellectual content.

## Funding

The article processing charge was funded by the German Research Foundation (DFG) and the Albert Ludwigs University Freiburg in the funding programme Open Access Publishing. This study was supported in part by the German Research Foundation (DFG; AL 1179/2-1, AL 1179/1-1, AR 341/5-1A).

### Conflict of interest statement

The authors declare that the research was conducted in the absence of any commercial or financial relationships that could be construed as a potential conflict of interest.
