# Supplementary material for: Enterococcus faecalis from Food, Clinical Specimens, and Oral Sites: Prevalence of Virulence Factors in Association with Biofilm Formation
Source: Front Microbiol. 2016 Jan 11;6:1534. doi: 10.3389/fmicb.2015.01534 (PMC4707231; doi:10.3389/fmicb.2015.01534)
Supplement: Supplementary file 1 [file DataSheet1.DOCX]

Supplementary Material

**Enterococcus faecalis from food, clinical specimens and oral sites: Prevalence of virulence factors in association with biofilm formation**

**Annette Carola Anderson^1^*, Daniel Jonas^2^, Ingrid Huber^3^, Lamprini Karygianni^1^, Johan Wölber^1^, Elmar Hellwig^1^, Nicole Arweiler^4^, Kirstin Vach^5^, Annette Wittmer^6^, Ali Al-Ahmad^1^**

^1^Department of Operative Dentistry and Periodontology, Center for Dental Medicine, Medical

Center, University of Freiburg, Germany

^2^Institute for Environmental Health Sciences and Hospital Infection Control, University of

Freiburg, Germany

^3^Bavarian Health and Food Safety Authority, Oberschleissheim, Germany

^4^Department of Periodontology, Philipps-University, Marburg, Germany

^5^Center for Medical Biometry and Medical Informatics, Institute for Medical Biometry and Statistics, Medical Center, University of Freiburg

^6^Institute for Microbiology and Hygiene, Medical Center, University of Freiburg, Germany

*** Correspondence:** Dr. Annette Carola Anderson, Department of Operative Dentistry and Periodontology, Center for Dental Medicine, Albert-Ludwigs-University, Hugstetter Strasse 55, 79106 Freiburg, Germany

Email: annette.anderson@uniklinik-freiburg.de

# Supplementary Figure

**Supplementary Figure 1A-F.** PFGE-patterns of *Enterococcus faecalis* from endodontic, food, clinical and plaque/saliva samples. Numbering corresponds to numbers in Table 3. R= reference strain.


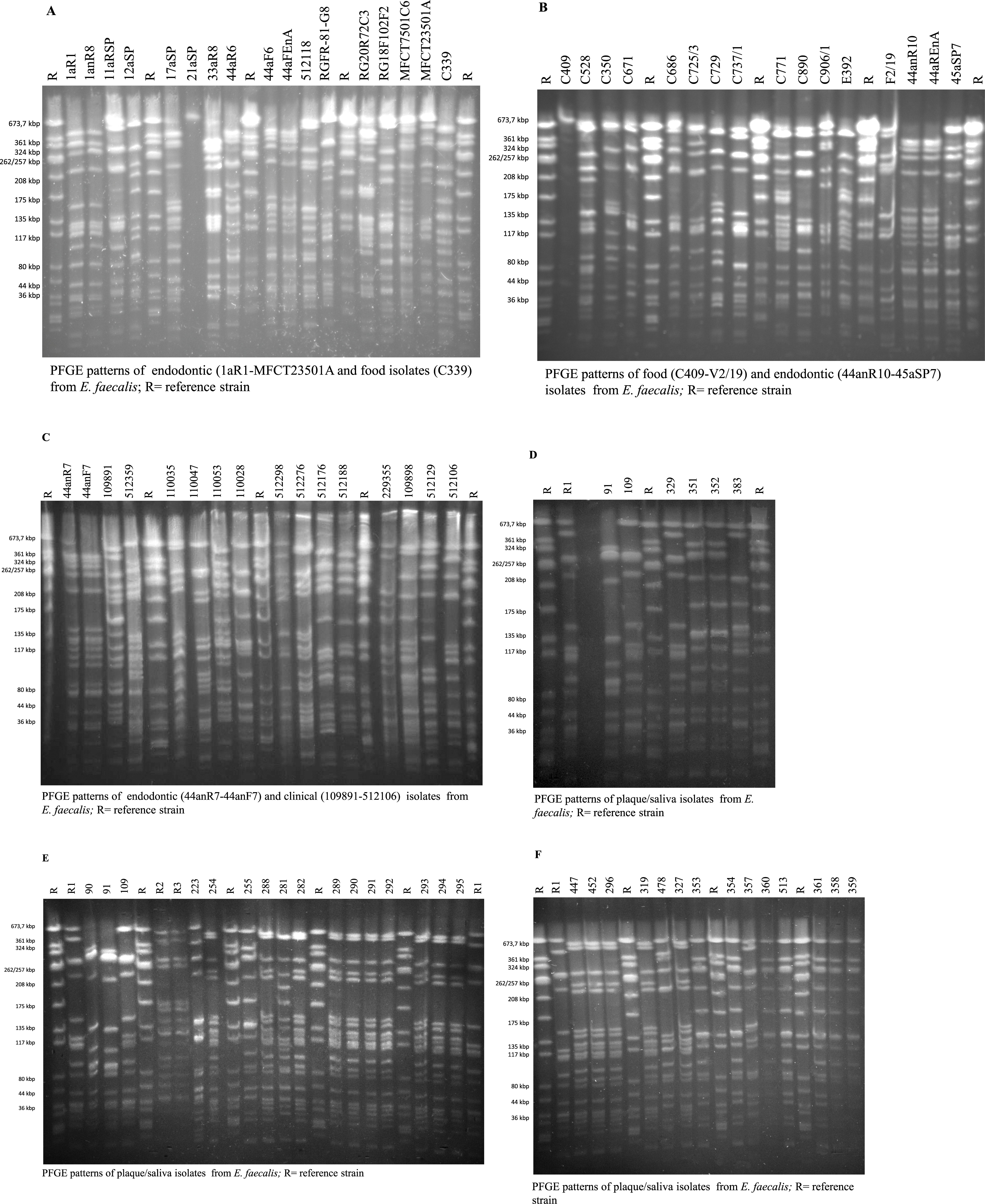


**Supplementary Table 1.** Antibiotic sensitivity of E. faecalis isolates from endodontic, plaque/saliva, food and clinical samples analyzed with VITEK (AST-P616 cards). MIC-values that are interpreted as resistance according to EUCAST standards are marked in bold, intermediate results in bold italics.

| Isolate/  Origin | Peni-  cillin G* | Ampi-  cillin | Ampi.-  Sulbactam | Imi-  penem | Erythro-  mycin | Levo-  floxacin | Cipro-  floxacin | Trimetho-  prim/Sulfa-methoxazol | Tetra-  cyclin | Tige-cycline | Nitro-  furantoin | Vanco-  mycin |
| --- | --- | --- | --- | --- | --- | --- | --- | --- | --- | --- | --- | --- |
| **Endodontic** |  |  |  |  |  |  |  |  |  |  |  |  |
| 1aR1 | 0.5 | ≤2 | ≤2 | ≤1 | **2** | 1 | ≤0.5 | ***≤10.0*** | 2 | ** | ** | ** |
| 1anR8 | 0.25 | ≤2 | ≤2 | ≤1 | **0.5** | 1 | 1 | ***≤10.0*** | 1 | ≤0.12 | ≤16 | 1 |
| 11aRSP | 8 | ≤2 | ≤2 | ≤1 | **0.5** | 1 | 1 | ***≤10.0*** | ≤1 | ≤0.12 | ≤16 | 1 |
| 12aSP | 2 | ≤2 | ≤2 | ≤1 | **4** | 1 | ≤0.5 | ***≤10.0*** | **≥16** | ≤0.12 | ≤16 | 1 |
| 17aSP | 2 | ≤2 | ≤2 | ≤1 | **2** | 2 | 1 | ***≤10.0*** | **≥16** | 0.25 | ≤16 | 1 |
| 21aSP | 4 | ≤2 | ≤2 | ≤1 | **2** | 1 | ≤0.5 | ***≤10.0*** | **≥16** | ≤0.12 | ≤16 | 2 |
| 33aR8 | 2 | ≤2 | ≤2 | ≤1 | **2** | 1 | 1 | ***≤10.0*** | ≤1 | ≤0.12 | ≤16 | 1 |
| 39aR6SP | 2 | ≤2 | ≤2 | ≤1 | **2** | 1 | ≤0.5 | ***≤10.0*** | 1 | ≤0.12 | ≤16 | 1 |
| 44aR6 | 4 | ≤2 | ≤2 | ≤1 | **2** | 1 | ≤0.5 | ***≤10.0*** | **≥16** | ≤0.12 | ≤16 | 1 |
| 44aREnA | 4 | ≤2 | ≤2 | ≤1 | **2** | 1 | ≤0.5 | ***≤10.0*** | **≥16** | ≤0.12 | ≤16 | 1 |
| 44aF6 | 4 | ≤2 | ≤2 | ≤1 | **2** | 2 | 1 | ***≤10.0*** | **≥16** | ≤0.12 | ≤16 | 1 |
| 44aFEnA | 4 | ≤2 | ≤2 | ≤1 | **2** | 2 | 1 | ***≤10.0*** | **≥16** | ≤0.12 | ≤16 | 1 |
| 44anR7 | 4 | ≤2 | ≤2 | ≤1 | **1** | 2 | 1 | ***≤10.0*** | **≥16** | ≤0.12 | ≤16 | 1 |
| 44anR10 | 4 | ≤2 | ≤2 | ≤1 | **1** | 1 | 1 | ***≤10.0*** | **≥16** | ≤0.12 | ≤16 | 1 |
| 44anF7 | 4 | ≤2 | ≤2 | ≤1 | **2** | 2 | 1 | ***≤10.0*** | **≥16** | ≤0.12 | ≤16 | 1 |
| 45aSP7 | 2 | ≤2 | ≤2 | ≤1 | **2** | 1 | 1 | ***≤10.0*** | 1 | ≤0.12 | ≤16 | 1 |
| 52F7 | 4 | ≤2 | ≤2 | ≤1 | **2** | 1 | 0.5 | ***≤10.0*** | 1 | ≤0.12 | ≤16 | 1 |
| 52R7 | 4 | ≤2 | ≤2 | ≤1 | **2** | 1 | 0.5 | ***≤10.0*** | 1 | ≤0.12 | ≤16 | 1 |
| 53F6 | 8 | ≤2 | ≤2 | ≤1 | **1** | 2 | 1 | ***≤10.0*** | 1 | ≤0.12 | ≤16 | 1 |
| 53R6 | 8 | ≤2 | ≤2 | 2 | **1** | 2 | ***2*** | ***≤10.0*** | 1 | ≤0.12 | ≤16 | 1 |
| 54F11 | 4 | ≤2 | ≤2 | ≤1 | **≥8** | 2 | 1 | ***≤10.0*** | 1 | ≤0.12 | ≤16 | 1 |
| 54QC6 | 4 | ≤2 | ≤2 | ≤1 | **≥8** | 2 | 1 | ***≤10.0*** | 1 | ≤0.12 | ≤16 | 1 |
| 55F7 | 4 | ≤2 | ≤2 | ≤1 | **2** | 1 | 0.5 | ***≤10.0*** | 1 | ≤0.12 | ≤16 | 2 |
| 55R6 | 1 | ≤2 | ≤2 | ≤1 | **1** | 1 | 0.5 | ***≤10.0*** | 1 | ≤0.12 | ≤16 | 2 |
| 57R9 | 2 | ≤2 | ≤2 | ≤1 | **1** | 2 | 1 | ***≤10.0*** | 2 | ≤0.12 | ≤16 | 2 |
| RGFR-81G8 | ** | ** | ** | ** | ** | ** | ** | ** | ** | ** | ** | ** |
| RG20R72C3 | ** | ** | ** | ** | ** | ** | ** | ** | ** | ** | ** | ** |
| RG18F102F2 | 4 | ≤2 | ≤2 | ≤1 | **≤0.25** | 1 | 0.5 | ***≤10.0*** | **≥16** | ≤0.12 | ≤16 | 2 |
| MFCT7501C6 | 2 | ≤2 | ≤2 | ≤1 | **2** | 1 | 0.5 | ***≤10.0*** | 1 | ≤0.12 | ≤16 | ≤0.5 |
| MFCT23501A1 | 2 | ≤2 | ≤2 | ≤1 | **2** | 1 | 1 | ***≤10.0*** | **≥16** | ≤0.12 | ≤16 | 2 |
| Isolate/  Origin | Peni-  cillin G* | Ampi-  cillin | Ampi.-  Sulbactam | Imi-  penem | Erythro-  mycin | Levo-  floxacin | Cipro-  floxacin | Trimetho-  prim/Sulfa-methoxazol | Tetra-  cyclin | Tige-cycline | Nitro-  furantoin | Vanco-  mycin |
| **Plaque/Saliva** |  |  |  |  |  |  |  |  |  |  |  |  |
| 90 | 4 | ≤2 | ≤2 | ≤1 | **4** | 2 | 1 | ***≤10.0*** | ≤1 | ≤0.12 | ≤16 | 1 |
| 91 | 4 | ≤2 | ≤2 | ≤1 | **4** | 2 | 1 | ***≤10.0*** | ≤1 | ≤0.12 | ≤16 | 1 |
| 223 | 2 | ≤2 | ≤2 | ≤1 | **≤0.25** | 1 | 1 | ***≤10.0*** | ≤1 | ≤0.12 | ≤16 | 1 |
| 254 | 8 | ≤2 | ≤2 | 2 | **≥8** | 2 | 1 | ***≤10.0*** | **≥16** | ≤0.12 | ≤16 | 1 |
| 255 | 4 | ≤2 | ≤2 | ≤1 | **2** | 1 | ≤0.5 | ***≤10.0*** | **≥16** | ≤0.12 | ≤16 | 2 |
| 281 | 2 | ≤2 | ≤2 | ≤1 | **2** | 2 | 1 | ***≤10.0*** | ≤1 | ≤0.12 | 64 | 2 |
| 282 | 8 | ≤2 | ≤2 | ≤1 | **≥8** | 2 | 1 | ***≤10.0*** | **≥16** | ≤0.12 | ≤16 | 1 |
| 288 | 8 | ≤2 | ≤2 | ≤1 | **≥8** | 1 | ≤0.5 | ***≤10.0*** | **≥16** | ≤0.12 | ≤16 | 1 |
| 289 | 8 | ≤2 | ≤2 | ≤1 | **≥8** | 1 | ≤0.5 | ***≤10.0*** | **≥16** | ≤0.12 | ≤16 | 1 |
| 290 | 8 | ≤2 | ≤2 | ≤1 | **≥8** | 1 | ≤0.5 | ***≤10.0*** | **≥16** | ≤0.12 | ≤16 | 1 |
| 291 | 8 | ≤2 | ≤2 | ≤1 | **≥8** | 1 | ≤0.5 | ***≤10.0*** | **≥16** | ≤0.12 | ≤16 | 1 |
| 292 | 8 | ≤2 | ≤2 | ≤1 | **≥8** | 1 | ≤0.5 | ***≤10.0*** | **≥16** | ≤0.12 | ≤16 | 1 |
| 293 | 8 | ≤2 | ≤2 | ≤1 | **≥8** | 1 | ≤0.5 | ***≤10.0*** | **≥16** | ≤0.12 | ≤16 | 1 |
| 294 | 8 | ≤2 | ≤2 | ≤1 | **≥8** | 1 | ≤0.5 | ***≤10.0*** | **≥16** | ≤0.12 | ≤16 | 1 |
| 295 | 8 | ≤2 | ≤2 | ≤1 | **≥8** | 1 | ≤0.5 | ***≤10.0*** | **≥16** | ≤0.12 | ≤16 | 1 |
| 296 | 8 | ≤2 | ≤2 | ≤1 | **≥8** | 1 | ≤0.5 | ***≤10.0*** | **≥16** | ≤0.12 | ≤16 | 1 |
| 319 | 8 | ≤2 | ≤2 | ≤1 | **≥8** | 1 | ≤0.5 | ***≤10.0*** | **≥16** | ≤0.12 | ≤16 | 1 |
| 327 | 8 | ≤2 | ≤2 | ≤1 | **≥8** | 1 | ≤0.5 | ***≤10.0*** | **≥16** | ≤0.12 | ≤16 | 1 |
| 351 | 4 | ≤2 | ≤2 | ≤1 | **2** | 1 | 1 | ***≤10.0*** | **≥16** | ≤0.12 | ≤16 | 2 |
| 352 | ** | ** | ** | ** | ** | ** | ** | ** | ** | ** | ** | ** |
| 353 | 2 | ≤2 | ≤2 | ≤1 | **2** | 1 | ≤0.5 | ***≤10.0*** | **≥16** | ≤0.12 | ≤16 | 2 |
| 354 | 2 | ≤2 | ≤2 | ≤1 | **2** | 1 | ≤0.5 | ***≤10.0*** | **≥16** | ≤0.12 | ≤16 | 2 |
| 357 | 2 | ≤2 | ≤2 | ≤1 | **≤0.25** | 1 | 1 | ***≤10.0*** | ≤1 | ≤0.12 | 32 | 1 |
| 358 | 2 | ≤2 | ≤2 | ≤1 | **2** | 1 | ≤0.5 | ***≤10.0*** | **≥16** | ≤0.12 | ≤16 | 2 |
| 359 | 2 | ≤2 | ≤2 | ≤1 | **2** | 1 | ≤0.5 | ***≤10.0*** | **≥16** | ≤0.12 | ≤16 | 2 |
| 360 | 8 | ≤2 | ≤2 | ≤1 | **2** | 2 | 1 | ***≤10.0*** | **≥16** | ≤0.12 | 32 | 2 |
| 361 | 4 | ≤2 | ≤2 | ≤1 | **2** | 1 | 1 | ***≤10.0*** | **≥16** | ≤0.12 | ≤16 | 2 |
| 383 | 4 | ≤2 | ≤2 | ≤1 | **2** | 2 | 1 | ***≤10.0*** | **≥16** | ≤0.12 | ≤16 | 2 |
| 446 | 8 | ≤2 | ≤2 | 2 | **≥8** | 2 | 1 | ***≤10.0*** | **≥16** | ≤0.12 | ≤16 | 1 |
| 447 | 8 | ≤2 | ≤2 | ≤1 | **≥8** | 1 | ≤0.5 | ***≤10.0*** | **≥16** | ≤0.12 | ≤16 | 1 |
| 448 | 8 | ≤2 | ≤2 | 2 | **≥8** | 2 | ≤0.5 | ***≤10.0*** | **≥16** | ≤0.12 | ≤16 | 1 |
| 449 | 8 | ≤2 | ≤2 | 2 | **≥8** | 1 | ≤0.5 | ***≤10.0*** | **≥16** | ≤0.12 | ≤16 | 1 |
| 450 | 8 | ≤2 | ≤2 | ≤1 | **≥8** | 1 | ≤0.5 | ***≤10.0*** | **≥16** | ≤0.12 | ≤16 | 1 |
| 451 | 8 | ≤2 | ≤2 | ≤1 | **≥8** | 1 | ≤0.5 | ***≤10.0*** | **≥16** | ≤0.12 | ≤16 | 1 |
| 452 | 8 | ≤2 | ≤2 | ≤1 | **≥8** | 1 | ≤0.5 | ***≤10.0*** | **≥16** | ≤0.12 | ≤16 | 1 |
| 478 | 2 | ≤2 | ≤2 | ≤1 | **2** | 1 | ≤0.5 | ***≤10.0*** | **≥16** | ≤0.12 | ≤16 | 2 |
| 513 | 8 | ≤2 | ≤2 | ≤1 | **2** | 2 | 1 | ***≤10.0*** | **≥16** | ≤0.12 | 32 | 2 |
| Isolate/  Origin | Peni-  cillin G* | Ampi-  cillin | Ampi.-  Sulbactam | Imi-  penem | Erythro-  mycin | Levo-  floxacin | Cipro-  floxacin | Trimetho-  prim/Sulfa-methoxazol | Tetra-  cyclin | Tige-cycline | Nitro-  furantoin | Vanco-  mycin |
| **Food** |  |  |  |  |  |  |  |  |  |  |  |  |
| F2/19 | 2 | ≤2 | ≤2 | ≤1 | **2** | 1 | ≤0.5 | ***≤10.0*** | **≥16** | ≤0.12 | 64 | 1 |
| E392 | 2 | ≤2 | ≤2 | ≤1 | **1** | 0.5 | ≤0.5 | ***≤10.0*** | **≥16** | ≤0.12 | ≤16 | 1 |
| C339 | 2 | ≤2 | ≤2 | ≤1 | **2** | 2 | 1 | ***≤10.0*** | **≥16** | ≤0.12 | ≤16 | 1 |
| C350 | 2 | ≤2 | ≤2 | ≤1 | **8** | 0.5 | ≤0.5 | ***≤10.0*** | **≥16** | ≤0.12 | ≤16 | 1 |
| C409 | 2 | ≤2 | ≤2 | ≤1 | **0.5** | 1 | ≤0.5 | ***≤10.0*** | **≥16** | ≤0.12 | ≤16 | 1 |
| C528 | 2 | ≤2 | ≤2 | ≤1 | **2** | 1 | ≤0.5 | ***≤10.0*** | ≤1 | ≤0.12 | ≤16 | 1 |
| C671 | 2 | ≤2 | ≤2 | ≤1 | **4** | 0.5 | ≤0.5 | ***≤10.0*** | **≥16** | ≤0.12 | ≤16 | 1 |
| C686 | 2 | ≤2 | ≤2 | ≤1 | **4** | 1 | ≤0.5 | ***≤10.0*** | **≥16** | ≤0.12 | ≤16 | 1 |
| C725/3 | 1 | ≤2 | ≤2 | ≤1 | **2** | 0.5 | ≤0.5 | ***≤10.0*** | **≥16** | ≤0.12 | ≤16 | 1 |
| C729 | 2 | ≤2 | ≤2 | ≤1 | **8** | 1 | 1 | **80** | **≥16** | ≤0.12 | 32 | 0.5 |
| C737/1 | 2 | ≤2 | ≤2 | ≤1 | **8** | 1 | 1 | **≥320** | **≥16** | ≤0.12 | ≤16 | 0.5 |
| C739 | ** | ** | ** | ** | ** | ** | ** | ** | ** | ** | ** | ** |
| C771 | 2 | ≤2 | ≤2 | ≤1 | **2** | 1 | 1 | ***≤10.0*** | **≥16** | ≤0.12 | ≤16 | 1 |
| C890 | 2 | ≤2 | ≤2 | ≤1 | **2** | 1 | ≤0.5 | ***≤10.0*** | **≥16** | ≤0.12 | 32 | 1 |
| C906/1 | 2 | ≤2 | ≤2 | ≤1 | **2** | 1 | 1 | ***≤10.0*** | **≥16** | ≤0.12 | ≤16 | 2 |
| Isolate/  Origin | Peni-  cillin G* | Ampi-  cillin | Ampi.-  Sulbactam | Imi-  penem | Erythro-  mycin | Levo-  floxacin | Cipro-  floxacin | Trimetho-  prim/Sulfa-methoxazol | Tetra-  cyclin | Tige-cycline | Nitro-  furantoin | Vanco-  mycin |
| **Clinical** |  |  |  |  |  |  |  |  |  |  |  |  |
| 110028 | 2 | ≤2 | ≤2 | ≤1 | **2** | 1 | 1 | ***≤10.0*** | **≥16** | ≤0.12 | ≤16 | 2 |
| 110035 | 4 | ≤2 | ≤2 | ≤1 | **≥8** | 2 | 1 | **80** | **≥16** | ≤0.12 | ≤16 | 1 |
| 110047 | 4 | ≤2 | ≤2 | ≤1 | **2** | 2 | 1 | ***≤10.0*** | ≤1 | ≤0.12 | ≤16 | 1 |
| 110053 | 8 | ≤2 | ≤2 | 2 | **≥8** | **≥8** | **≥8** | **≥320** | **≥16** | ≤0.12 | ≤16 | 1 |
| 109891 | 8 | ≤2 | ≤2 | 2 | **≥8** | **≥8** | **≥8** | **≥320** | **≥16** | ≤0.12 | ≤16 | 1 |
| 109898 | 2 | ≤2 | ≤2 | ≤1 | **8** | 1 | ≤0.5 | ***≤10.0*** | **≥16** | ≤0.12 | ≤16 | 1 |
| 229355 | 2 | ≤2 | ≤2 | ≤1 | **2** | 2 | ≤0.5 | ***≤10.0*** | **≥16** | ≤0.12 | ≤16 | 2 |
| 512106 | 1 | ≤2 | ≤2 | ≤1 | **4** | 2 | 1 | ***≤10.0*** | **≥16** | ≤0.12 | ≤16 | 1 |
| 512118 | 4 | ≤2 | ≤2 | ≤1 | **≥8** | 1 | 1 | **≥320** | **≥16** | ≤0.12 | ≤16 | 1 |
| 512129 | 8 | ≤2 | ≤2 | ≤1 | **1** | 1 | ≤0.5 | ***≤10.0*** | **≥16** | ≤0.12 | ≤16 | 1 |
| 512176 | 32 | ≤2 | ≤2 | 4 | **≥8** | **≥8** | **≥8** | **≥320** | ≤1 | ≤0.12 | ≤16 | 1 |
| 512188 | 16 | ≤2 | ≤2 | 4 | **≥8** | **≥8** | **≥8** | **≥320** | **≥16** | ≤0.12 | ≤16 | 1 |
| 512276 | 4 | ≤2 | ≤2 | ≤1 | **≥8** | 1 | ≤0.5 | ***≤10.0*** | **≥16** | ≤0.12 | ≤16 | 1 |
| 512298 | 4 | ≤2 | ≤2 | ≤1 | **2** | 2 | 1 | ***≤10.0*** | **≥16** | ≤0.12 | ≤16 | 2 |
| 512359 | 2 | ≤2 | ≤2 | ≤1 | **≥8** | **≥8** | **≥8** | **≥320** | **≥16** | ≤0.12 | ≤16 | 1 |
| Reference strains |  |  |  |  |  |  |  |  |  |  |  |  |
| *E. faecalis* 12030 | 4 | ≤2 | ≤2 | ≤1 | **0.5** | 1 | 1 | ***≤10.0*** | **≥16** | ≤0.12 | ≤16 | 1 |
| *E. faecium* 137 | ≥64 | **≥32** | **≥32** | **≥16** | **≥8** | **≥8** | **≥8** | **≥320** | **≤1** | ≤0.12 | - | **≥32** |

| Isolate/  Origin | Teico-  planin | Line-  zolid | Gentamicin  High  level |
| --- | --- | --- | --- |
| **Endodontic** |  |  |  |
| 1aR1 | ** | 4 | ** |
| 1anR8 | ≤0.5 | 2 | Syn-S*** |
| 11aRSP | ** | ** | ** |
| 12aSP | ≤0.5 | 2 | Syn-S |
| 17aSP | ≤0.5 | **≥8** | Syn-S |
| 21aSP | ≤0.5 | 2 | Syn-S |
| 33aR8 | ≤0.5 | 2 | Syn-S |
| 39aR6SP | ≤0.5 | 2 | Syn-S |
| 44aR6 | ≤0.5 | 2 | Syn-S |
| 44aREnA | ≤0.5 | 1 | Syn-S |
| 44aF6 | ≤0.5 | 2 | Syn-S |
| 44aFEnA | ≤0.5 | 1 | Syn-S |
| 44anR7 | ≤0.5 | 1 | Syn-S |
| 44anR10 | ≤0.5 | 1 | Syn-S |
| 44anF7 | ≤0.5 | 1 | Syn-S |
| 45aSP7 | ≤0.5 | 2 | Syn-S |
| 52F7 | ≤0.5 | 2 | Syn-S |
| 52R7 | ≤0.5 | 2 | Syn-S |
| 53F6 | ≤0.5 | 2 | Syn-S |
| 53R6 | ≤0.5 | 4 | Syn-S |
| 54F11 | ≤0.5 | 2 | Syn-S |
| 54QC6 | ≤0.5 | 2 | Syn-S |
| 55F7 | ≤0.5 | 2 | Syn-S |
| 55R6 | ≤0.5 | 2 | Syn-S |
| 57R9 | ≤0.5 | 2 | Syn-S |
| RGFR-81G8 | ** | ** | ** |
| RG20R72C3 | ** | ** | ** |
| RG18F102F2 | ≤0.5 | 2 | Syn-S |
| MFCT7501C6 | ≤0.5 | 2 | Syn-S |
| MFCT23501A1 | ≤0.5 | 2 | Syn-S |
| Isolate/  Origin | Teico-  planin | Line-  zolid | Gentamicin  High  level |
| **Plaque/Saliva** |  |  |  |
| 90 | ≤0.5 | 2 | Syn-S |
| 91 | ≤0.5 | 2 | Syn-S |
| 223 | ≤0.5 | 2 | Syn-S |
| 254 | ≤0.5 | 2 | **Syn-R** |
| 255 | ≤0.5 | 2 | Syn-S |
| 281 | ≤0.5 | 2 | Syn-S |
| 282 | ≤0.5 | 2 | **Syn-R** |
| 288 | ≤0.5 | 1 | **Syn-R** |
| 289 | ≤0.5 | 2 | **Syn-R** |
| 290 | ≤0.5 | 2 | **Syn-R** |
| 291 | ≤0.5 | 2 | **Syn-R** |
| 292 | ≤0.5 | 2 | **Syn-R** |
| 293 | ≤0.5 | 2 | **Syn-R** |
| 294 | ≤0.5 | 2 | **Syn-R** |
| 295 | ≤0.5 | 2 | **Syn-R** |
| 296 | ≤0.5 | 2 | **Syn-R** |
| 319 | ≤0.5 | 2 | **Syn-R** |
| 327 | ≤0.5 | 2 | **Syn-R** |
| 351 | ≤0.5 | 2 | Syn-S |
| 352 | ** | ** | ** |
| 353 | ≤0.5 | 2 | Syn-S |
| 354 | ≤0.5 | 2 | Syn-S |
| 357 | ≤0.5 | 2 | Syn-S |
| 358 | ≤0.5 | 2 | Syn-S |
| 359 | ≤0.5 | 2 | Syn-S |
| 360 | ≤0.5 | 2 | Syn-S |
| 361 | ≤0.5 | 2 | Syn-S |
| 383 | ≤0.5 | 4 | Syn-S |
| 446 | ≤0.5 | 2 | **Syn-R** |
| 447 | ≤0.5 | 2 | **Syn-R** |
| 448 | ≤0.5 | 2 | **Syn-R** |
| 449 | ≤0.5 | 2 | **Syn-R** |
| 450 | ≤0.5 | 2 | **Syn-R** |
| 451 | ≤0.5 | 2 | **Syn-R** |
| 452 | ≤0.5 | 2 | **Syn-R** |
| 478 | ≤0.5 | 2 | Syn-S |
| 513 | ≤0.5 | 2 | Syn-S |
| Isolate/  Origin | Teico-  planin | Line-  zolid | Gentamicin  High  level |
| **Food** |  |  |  |
| F2/19 | ≤0.5 | 2 | Syn-S |
| E392 | ≤0.5 | 2 | Syn-S |
| C339 | ≤0.5 | 2 | Syn-S |
| C350 | ≤0.5 | 2 | Syn-S |
| C409 | ≤0.5 | 2 | Syn-S |
| C528 | ≤0.5 | 2 | Syn-S |
| C671 | ≤0.5 | 2 | Syn-S |
| C686 | ≤0.5 | 2 | Syn-S |
| C725/3 | ≤0.5 | 2 | Syn-S |
| C729 | ≤0.5 | 2 | Syn-S |
| C737/1 | ≤0.5 | 2 | Syn-S |
| C739 | ** | ** | ** |
| C771 | ≤0.5 | 2 | Syn-S |
| C890 | ≤0.5 | 2 | Syn-S |
| C906/1 | ≤0.5 | 2 | Syn-S |
| Isolate/  Origin | Teico-  planin | Line-  zolid | Gentamicin  High  level |
| **Clinical** |  |  |  |
| 110028 | ≤0.5 | 4 | Syn-S |
| 110035 | ≤0.5 | 2 | **Syn-R** |
| 110047 | ≤0.5 | 2 | Syn-S |
| 110053 | ≤0.5 | 2 | **Syn-R** |
| 109891 | ≤0.5 | 2 | **Syn-R** |
| 109898 | ≤0.5 | 2 | **Syn-R** |
| 229355 | ≤0.5 | 2 | Syn-S |
| 512106 | ≤0.5 | 2 | Syn-S |
| 512118 | ≤0.5 | 2 | Syn-S |
| 512129 | ≤0.5 | 2 | Syn-S |
| 512176 | ≤0.5 | 2 | **Syn-R** |
| 512188 | 1 | 2 | **Syn-R** |
| 512276 | ≤0.5 | 2 | **Syn-R** |
| 512298 | ≤0.5 | 2 | Syn-S |
| 512359 | ≤0.5 | 2 | **Syn-R** |
| Reference strains |  |  |  |
| *E. faecalis* 12030 | ≤0.5 | 2 | Syn-S |
| *E. faecium* 137 | **≥32** | 2 | Syn-S |

*insufficient empirical evidence for therapy of this species with this antibiotic

**test not possible due to insufficient growth

*** synergy sensitive, synergy resistant, resp.
